# Mercury in fish and adverse reproductive outcomes: results from South Carolina

**DOI:** 10.1186/1476-072X-13-30

**Published:** 2014-08-15

**Authors:** James B Burch, Sara Wagner Robb, Robin Puett, Bo Cai, Rebecca Wilkerson, Wilfried Karmaus, John Vena, Erik Svendsen

**Affiliations:** 1 Department of Epidemiology & Biostatistics, Arnold School of Public Health, University of South Carolina, Columbia, SC, USA; 2 WJB Dorn Veteran’s Affairs Medical Center, Columbia, SC, USA; 3 College of Public Health, Department of Epidemiology and Biostatistics, University of Georgia, Athens, GA, USA; 4 Maryland Institute for Applied Environmental Health, University of Maryland, College Park, MD, USA; 5 Institute for Families in Society, University of South Carolina, Columbia, SC, USA; 6 Division of Epidemiology, Biostatistics, and Environmental Health, School of Public Health, University of Memphis, Memphis, TN, USA; 7 Department of Public Health Sciences, Medical University of South Carolina, Charleston, SC, USA; 8 Department of Global Environmental Health Sciences, Tulane University School of Public Health and Tropical Medicine, New Orleans, LA, USA

**Keywords:** Low birth weight, Environmental public health tracking, Geographic information system, Preterm birth

## Abstract

**Background:**

Mercury is a metal with widespread distribution in aquatic ecosystems and significant neurodevelopmental toxicity in humans. Fish biomonitoring for total mercury has been conducted in South Carolina (SC) since 1976, and consumption advisories have been posted for many SC waterways. However, there is limited information on the potential reproductive impacts of mercury due to recreational or subsistence fish consumption.

**Methods:**

To address this issue, geocoded residential locations for live births from the Vital Statistics Registry (1995–2005, N = 362,625) were linked with spatially interpolated total mercury concentrations in fish to estimate potential mercury exposure from consumption of locally caught fish. Generalized estimating equations were used to test the hypothesis that risk of low birth weight (LBW, <2,500 grams) or preterm birth (PTB, <37 weeks clinical gestation) was greater among women living in areas with elevated total mercury in fish, after adjustment for confounding. Separate analyses estimated term LBW and PTB risks using residential proximity to rivers with fish consumption advisories to characterize exposure.

**Results:**

Term LBW was more likely among women residing in areas in the upper quartile of predicted total mercury in fish (odds ratio [OR] = 1.04; 95% confidence interval [CI]: 1.00-1.09) or within 8 kilometers of a river with a ‘do not eat’ fish advisory (1.05; 1.00-1.11) compared to the lowest quartile, or rivers without fish consumption restrictions, respectively. When stratified by race, risks for term LBW or PTB were 10-18% more likely among African-American (AA) mothers living in areas with the highest total fish mercury concentrations.

**Conclusions:**

To our knowledge, this is the first study to examine the relationship between fish total mercury concentrations and adverse reproductive outcomes in a large population-based sample that included AA women. The ecologic nature of exposure assessment in this study precludes causal inference. However, the results suggest a need for more detailed investigations to characterize patterns of local fish consumption and potential dose–response relationships between mercury exposure and adverse reproductive outcomes, particularly among AA mothers.

## Background

Low birth weight (LBW) and preterm birth (PTB) are important predictors of infant morbidity and mortality, and they contribute to a spectrum of long-term disabilities and chronic disease [1-3]. The prevalence and impacts of these adverse reproductive outcomes are magnified in the southeastern United States (US). In 2012, South Carolina (SC) ranked 3rd highest for LBW prevalence among the 50 US states (~10% versus the national rate of 9.0%) [4]. In 2011, SC also ranked poorly for PTB, with a prevalence of approximately 14% compared to a national value of 12% [4,5]. There are significant racial disparities in LBW and PTB both nationally and within SC [6-8]. In 2000, LBW prevalence among African-American (AA) women in SC (14.2%) was about twice that observed among European-American (EA) women (7.2%), and PTB prevalence among AA women (14.1%) was 47% higher than EA women (9.6%) [9]. Risk factors for LBW and PTB include a complex array of behavioral, social, genetic and environmental factors, and reasons for the notable racial disparity are not completely understood [10,11]. In SC, risk factors for LBW or PTB among AA mothers may include neighborhood contextual factors that contribute to maternal stress, such as poverty and inadequate access to social support networks [12,13]. However, environmental factors also may contribute to the role of maternal place of residence as a mediator of maternal stress and adverse reproductive outcomes among AA women [11,14,15].

Mercury is a widely distributed contaminant in aquatic ecosystems and it can elicit neurological and reproductive toxicity in humans with elevated exposure. A primary mechanism for its introduction into aquatic ecosystems is via atmospheric deposition from coal combustion sources. Atmospheric residence times for mercury can range from months up to a year, and thus atmospheric mercury releases can achieve a global distribution [16]. Transformation of inorganic mercury to methylmercury in sediments facilitates its bioaccumulation in aquatic species [16]. In 2009, a study of chemical residues in a representative sample of freshwater fish from lakes in the contiguous US indicated that mercury was present in 100% of the fish sampled, and that 49% exceeded the human health screening value for mercury (0.3 ppm fish criterion value) [17,18]. Fish consumption is a major human exposure pathway for mercury; hair or blood concentrations are higher among those who consume more fish relative to those with infrequent fish consumption [19-24]. The developing fetus is particularly susceptible to mercury toxicity. Numerous birth cohorts and other studies have characterized neurodevelopmental impacts associated with prenatal mercury exposure from maternal fish consumption [25-32]. However, inconsistent and in some cases unexpected protective effects have been associated with prenatal mercury exposure [30]. Fish consumption during pregnancy is an important source of protein, omega-3 fatty acids and other nutrients that benefit the developing fetus [25,26,33,34]. Balancing the beneficial developmental effects of fish consumption with the detrimental effects of mercury or other contaminant intake represents both a significant source of uncertainty for mercury-related health research, and an important consideration for public health interventions related to mercury exposure [34,35]. Results from studies examining the relationship between mercury exposure and LBW, PTB, or other reproductive outcomes suggest that adverse effects may be encountered, although inconsistencies also have been observed, indicating a need for more research [36-48]. Furthermore, the extent to which recreational or subsistence fishing may contribute to racial disparities in adverse reproductive outcomes has not been thoroughly examined. Fish consumption is consistently greater among AAs relative to EAs both in SC and throughout the US [21,22,49-54]. In two nationally representative studies, AA women of reproductive age had higher blood mercury concentrations compared with EA women [21,22]. A recent survey of fish consumption and fish mercury levels among AA women in a southeast US coastal community (Newport News, VA) indicated that 58-73% had estimated daily mercury intakes that exceeded the federal guideline (Reference dose [RfD]: 0.1 μg mercury/kg body weight per day), and that 2% exceeded an intake expected to elicit adverse neurodevelopmental effects in children (1.0 μg/kg-day or 10× the RfD) [50]. These findings suggest that even though AA women in some southeast US communities may not be subsistence fishers, they may have elevated subsistence fish consumption. To our knowledge, the relationship between mercury in fish and adverse reproductive outcomes has not been examined in a population of AA women.

The current study used an environmental public health tracking (EPHT) framework [55] to evaluate the relationship between total mercury concentrations in fish and adverse reproductive outcomes among SC women. This was achieved through linkage of existing public health and environmental data to test the hypothesis that women residing in areas with elevated fish total mercury concentrations have increased odds of delivering a term LBW or PTB infant. South Carolina has conducted total mercury biomonitoring in both fresh- and saltwater fish species since 1976, and data from this program are used to develop and distribute statewide fish consumption advisories for lakes and rivers [16]. Geostatistical modeling within a geographic information system (GIS) was used to characterize the spatial distribution of fish total mercury concentrations in SC, and those exposure estimates were combined with the latitude and longitude of birth residence using data from the SC Vital Statistics Birth Registry over an eleven year period (1995–2005). Once the data linkage was performed, personal identifying data were removed, allowing for examination of term LBW and PTB risk estimates in relation to estimated total mercury levels in fish. The study was reviewed and approved by University of South Carolina (USC) and South Carolina Department of Health and Environmental Control (DHEC) Institutional Review Boards.

## Methods

The study population was comprised of all live births in SC between 1995 and 2005. De-identified birth outcome data were obtained from the SC Department of Health and Environmental Control (DHEC) Office of Vital Statistics along with reproductive and demographic information. Term LBW cases were defined as full term babies less than 2,500 grams (5 lbs, 8 oz), and PTB was defined as births with less than 37 weeks of clinical gestation. Concurrent births (e.g., twins, triplets) and infants delivered via Cesaearian section were excluded. Nearly all residences of mothers having live births during the study period were geocoded in the SC birth registry, and only those geocoded with a Tier 5 matching accuracy (i.e., matched to the exact address, 84%) were included in the analysis. Other data obtained from birth certificates included race, adequacy of prenatal care (Kotelchuck index: defined as inadequate, intermediate, adequate, or adequate plus prenatal care), number of previous live births and stillborn infants, and maternal: age, smoking status, and education (11^th^ grade or less; 12^th^ grade, high school degree or general education degree [GED]; college). Hispanic women were excluded due to sparse data in some regions.

**Figure 1:**
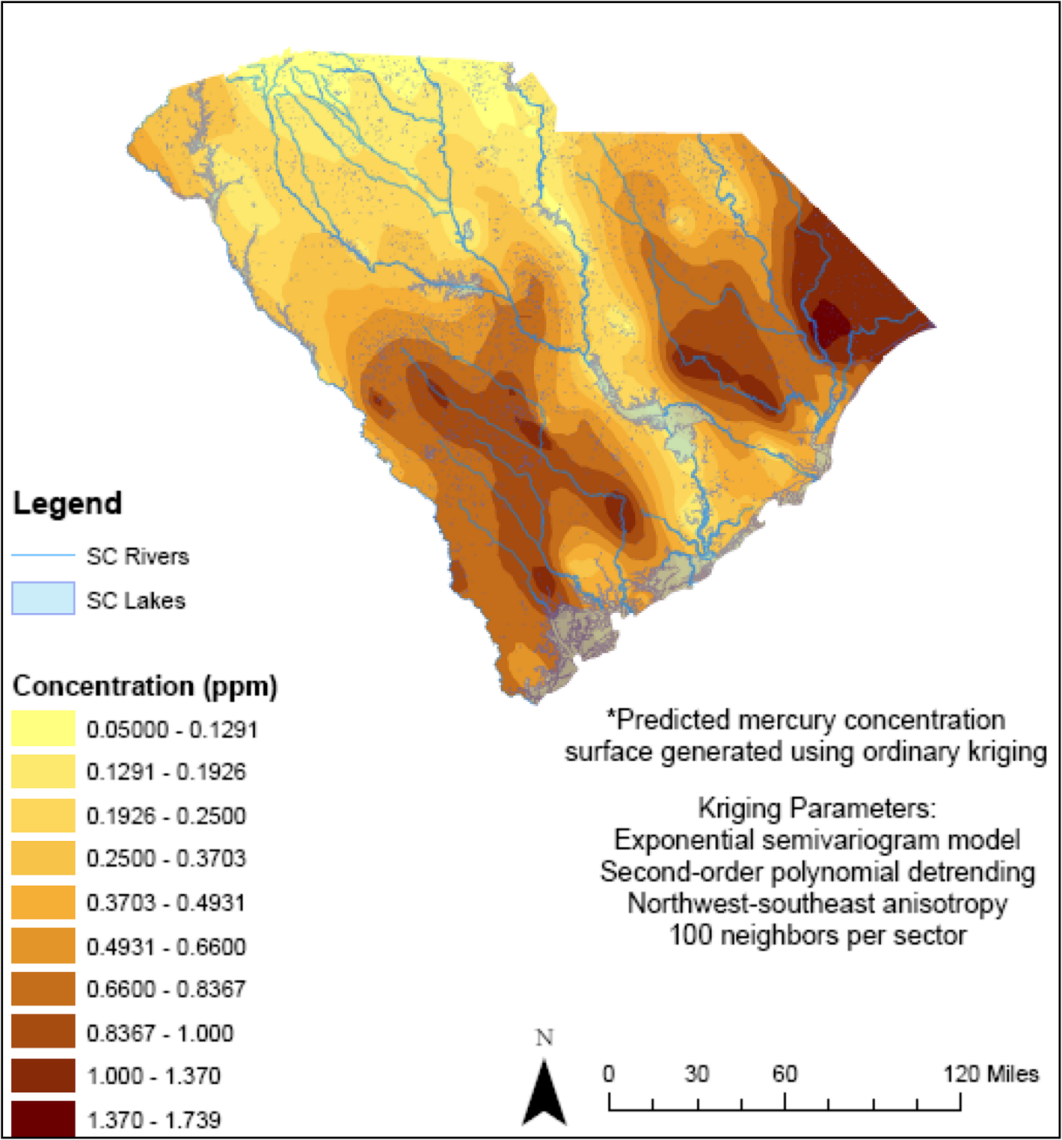
Predicted total mercury concentrations based on fish tissue samples*. Spatial interpolation of total mercury concentrations in South Carolina largemouth bass (1995–2005, N = 3,828 samples) obtained via ordinary kriging with the following model specifications: a U-shaped trend was modeled via a second-order polynomial; the semivariogram was fitted with an exponential model; anisotropy was assigned from northwest to southeast following the flow of water in SC; the searching neighborhood was defined as a directional ellipse with four sectors (100 neighbors per sector, minimum = 5). Gray areas correspond to southern coastal plain; there are no largemouth bass in these areas.

Concentrations of total fish mercury were obtained from the SC DHEC Bureau of Water for 1995–2005 [16]. The SC fish biomonitoring program follows a standardized protocol that includes quantification of fish total mercury concentrations by cold vapor atomic absorption spectrometry (EPA method 245.6; a program description is provided in [16]). Fish sampling targeted large public water bodies throughout SC; sampling criteria included consideration of public access, fishing opportunities, and successful catches. Smaller rivers and streams and private lakes were not sampled due to resource constraints. Largemouth bass data were used in the analysis as representative fish that are caught for consumption (N = 3,828 samples). Mercury levels in largemouth bass rank among the highest relative to other fish in SC, and they compare reasonably well with some but not all other commonly consumed species [16]. They are ubiquitous in SC freshwater and were well represented in the SC DHEC Bureau of Water database. Using spatial coordinates for fish sample locations, a geostatistical model (spatial interpolation via ordinary kriging) within a GIS (ArcGIS, Redlands, CA) was used to map the predicted statewide distribution of total mercury concentrations in fish (Figure 1). Kriging provides a statistically unbiased method for predicting unknown values from observed data at specific geographic locations [56]. Several parameters were evaluated to obtain the best fitting model of fish total mercury concentrations, including trend analysis, semivariogram model goodness-of-fit, and variations in the searching neighborhood. Trend analysis identified a U-shaped trend that was modeled with a second-order polynomial. The semivariogram was fitted with a mathematical model for use as an interpolator; spherical and exponential models were evaluated, and the Q-Q plot for the exponential model provided a better fit. Anisotropy from northwest to southeast, following the flow of water in SC, was used due to impacts on the semivariogram and the resulting fitted model. The searching neighborhood used to enclose measured points and predict values was defined with a directional ellipse with four sectors. The Geostatistical Wizard function in ArcGIS was used to evaluate how the shape and number of sectors affected the predicted value, as well as evaluate which neighbors were weighted more heavily to predicted values. To increase the statistical power of prediction, the number of neighbors per sector was increased to 100 (minimum: 5). Validation of the prediction model was performed with the ArcGIS Geostatistical Analyst tool using a 5% random sample of the data. The mean of the predicted errors (0.001815) and the Root-Mean-Square Standardized error (0.9289) were close to the optimal values 0 and 1, respectively; the slope of a plot of predicted values versus the measured mercury values was 0.771. The final geostatistical model was used to assign interpolated fish total mercury levels at each birth residence using the geocoded address (within ±0.005 ppm of the interpolated value at each location). The residence at birth was used to assign exposure. If a woman changed her residence between eligible births, the new location was used to assign exposure. Predicted fish mercury values at each birth residence were categorized into quartiles that were subsequently used in the statistical analysis to estimate term LBW or PTB risk. In separate analyses, term LBW or PTB was assessed using a categorical variable that grouped residences based on SC’s established fish advisory cut-points (<0.25 ppm, 0.25-0.66 ppm, 0.67-0.99 ppm, ≥1 ppm; using the predicted fish total mercury concentrations) [16]. These cut-points were derived from guidance developed for the US Great Lakes region using the US EPA RfD (0.1 μg mercury/kg body weight per day) and estimates of maximum daily mercury ingestion from fish (http://www.scdhec.gov/FoodSafety/FishConsumptionAdvisories[16,57]). Finally, an exposure variable was assigned to the birth residence based on its proximity to rivers with posted mercury fish consumption advisories (within or beyond 8 kilometers [5 miles]). To do this, the GIS was used to create a statewide map defining 8-km zones adjacent to major rivers and lakes according to each type of fish consumption advisory for mercury in largemouth bass (no restrictions, 1 meal/week, 1 meal/month, do not eat, not sampled), and potential exposure to total mercury in fish was assigned to each birth residence based on its location within or outside a given zone. Analyses of term LBW and PTB risk were performed using the type of advisory assigned to each birth residence as the exposure estimate for mercury in fish. This approach was implemented in an effort to characterize highly exposed subpopulations comprised of subsistence fishers and their families who may tend to fish in waterways close to their residence (within 8 km).

Data analyses were performed using the SAS (statistical analysis software) program (version 9.2, SAS Institute: Cary, NC). The relationship between term LBW or PTB and each total mercury exposure estimate was evaluated using generalized estimating equations (GENMOD procedure) with an unstructured correlation matrix. This procedure accommodates repeated measures and thus accounted for multiple live births (if present) occurring at different times for a given mother. Variables obtained from the vital registry (mother’s: race/ethnicity, age, education, reported smoking status during pregnancy, and number of previous live births and stillborns) were evaluated for potential confounding using a two-stage process. First, univariate associations with term LBW or PTB were identified (p <0.05). Variables selected in this manner were then included as covariates in a multivariable statistical model, and manual hierarchical backwards elimination was used to select variables for inclusion in the final model. Variables were retained if their removal from the model resulted in a change of at least 10% in the effect estimate for total mercury exposure. Odds ratios (ORs) with corresponding two-tailed 95% confidence intervals (CIs) were calculated to characterize the relationship between estimated fish total mercury exposure and each outcome, after adjustment for selected confounding factors (mother’s age, education, race, smoking status, number of previous live births and stillborns). Aikaike’s Information Criterion (AIC) and log likelihood tests were used to assess the goodness-of-fit of each multivariable model, and to evaluate the presence of confounding or effect modification. The Kotelchuck index was considered an intervening variable since mercury neurotoxicity might influence a women’s motivation, educational attainment, ability to reason, or knowledge of the benefits of prenatal care (p <0.05 via Sobel test). This index also was strongly associated with other independent variables in the analysis and modified the relationship between potential total mercury exposures and birth outcome; it was thus evaluated by stratification with race. Effect modification by maternal education and race also was evaluated via stratification. Ancillary analyses evaluated whether there were regional differences in the relationship between fish total mercury and term LBW or PTB. To do this, SC counties were assigned to the: Southern Plain, Mid-Atlantic Coastal Plain, or Coastal areas that generally overlap with SC ecoregions [16] except that the coastal region included all counties bordering the Atlantic ocean. The ‘upstate’ regions (Piedmont, Blue Ridge) were excluded due to low fish total mercury levels in those areas. The interaction between total mercury exposure and birth year was tested to evaluate possible differences in the effect over time. Finally, ancillary analyses were performed using unconditional multiple logistic regression in a subset of observations containing only outcomes for mother’s first birth (LBW N = 150,988; PTB N = 150,253); those analyses yielded results generally consistent with the main analysis except that risk estimates were attenuated in some cases (Additional file 1: Table S1).

## Results

The analytical dataset contained 362,625 live births after inclusion and exclusion criteria were applied. Most mothers were of non-Hispanic, EA race/ethnicity (61%), had a college degree (44%), no previous births (42%), and were non-smokers during their pregnancy (86%, Table 1). Most mothers also received adequate (39%) or adequate-plus (34%) prenatal care according to Kotelchuck index categories (Table 1). The average rates of term LBW and PTB for all mothers in this population-based sample throughout the eleven year study period were 7% and 9%, respectively (Table 1).

**Table 1:** Descriptive characteristics of mothers and live births, South Carolina, 1995–2005 (N = 362,625*)

**Characteristic**	**N (%)**
Mothers education	
11^th^ grade or less	81,797 (23)
12^th^ grade, high school diploma, GED	119,568 (33)
College/degree	159,346 (44)
Race/ethnicity	
European American, non-Hispanic	219,413 (61)
African American, non-Hispanic	119,461 (33)
Hispanic/other	23,706 ( 7)
Prenatal care (Kotelchuk Index)	
Inadequate	54,479 (15)
Intermediate	44,768 (13)
Adequate	137,930 (39)
Adequate plus	119,645 (34)
Smoking status during pregnancy	
Smoker	49,643 (14)
Non-smoker	312,376 (86)
Low birth weight	
<2,500 grams	25,354 (7)
≥2,500 grams	337,236 (93)
Preterm birth	
<37 weeks clinical gestation age	31,032 (9)
≥37 weeks clinical gestation age	329,680 (91)
Number of previous live births and stillborns	
0	152,154 (42)
1	121,306 (33)
≥2	88,793 (25)
Mother’s age, years (median [range])	25 (11–51)

Percentages totaling to less than 100% are due to missing values *Exclusions include: Cesarean births, concurrent births, and missing outcome data. GED: general equivalency diploma.

Compared to mothers with normal birth weight babies, women with term LBW infants were more likely to live in an area with higher predicted total mercury levels in fish (quartile 3 [Q3] OR: 1.06; 95% CI: 1.02-1.10; Q4 OR: 1.04; 95% CI: 1.00-1.09; Table 2). An increased odds of term LBW also was observed among women living within eight kilometers of a river with a ‘1-meal/month’ (OR: 1.09; 95% CI: 1.03-1.16) or ‘do not eat’ fish advisory for mercury (OR: 1.05; 95% CI: 1.00-1.11; Table 2). PTB infants were 9 percent more likely to have birth residences with estimated fish mercury exposures in the 2^nd^ (OR: 1.09; 95% CI: 1.06-1.13) or 3^rd^ (OR: 1.09; 95% CI: 1.05-1.13) highest exposure quartile (but not the 4^th^ quartile: Q4 OR: 1.02; 95% CI: 0.98-1.06) compared to those with low estimated fish mercury levels (Table 2). No other statistically significant increased odds of PTB were observed in this main analysis, and reduced odds of PTB in relation to estimated total mercury exposure were noted in two circumstances (Table 2).

**Table 2:** **Relationship between adverse reproductive outcomes and predicted mercury exposure, all live births, South Carolina, 1995-2005**^**a**^

**Exposure estimate**	**Low birth weight (N = 359,804)**		**Preterm birth (N = 357,996)**	
	**Odds ratio**	**95% Confidence interval**	**Odds ratio**	**95% Confidence interval**
Predicted Mercury in Fish^b^				
Quartile 1 (≤0.17 ppm)	Ref	-	Ref	-
Quartile 2 (>0.17-0.29 ppm)	1.03	0.99, 1.07	1.09	1.06, 1.13
Quartile 3 (>0.29-0.62 ppm)	1.06	1.02, 1.10	1.09	1.05, 1.13
Quartile 4 (>0.62 ppm)	1.04	1.00, 1.09	1.02	0.98, 1.06
Fish Advisory Categories				
<0.25 ppm	Ref	-	Ref	-
0.25-0.66 ppm	1.03	1.00, 1.07	1.03	1.00, 1.06
0.67-0.99 ppm	1.02	0.98, 1.06	0.96	0.93, 0.99
≥1.0 ppm	1.06	0.99, 1.14	1.03	0.97, 1.10
8-Kilometer Buffer Zones				
No restrictions	Ref	-	Ref	-
1 meal a week	0.95	0.91, 1.00	1.01	0.97, 1.05
1 meal a month	1.09	1.03, 1.16	0.86	0.82, 0.91
Do not eat	1.05	1.00, 1.11	0.97	0.92, 1.01

^a^Adjusted for: mother’s age, race, education, smoking status, and number of previous live births and stillborns ^b^Based on kriged interpolation model (see Figure 1) ppm: parts per million. N’s are smaller than those presented in Table 1 due to missing data). ND - below the limit of detection.

The p-values for interaction of each mercury exposure grouping with race, race by Kotelchuck index, or race by maternal education were all ≤0.07 (Type III sums of squares) for either LBW or PTB. When the analyses were stratified by race, term LBW among AA mothers was 1.04 to 1.18 times more likely to occur among infants whose mother resided in an area with elevated fish total mercury concentrations. Statistically significant increases in term LBW risk estimates were observed among AA mothers using exposures that were estimated via predicted fish total mercury levels, fish consumption advisories, or the 8-km buffer zones (Table 3). Results for PTB among AA mothers were similar to those obtained for term LBW when exposures were assigned using predicted fish total mercury concentrations at the residence, but not when exposures were estimated based on the proximity to rivers with fish consumption advisories (Table 3). There was no evidence for an increased risk of term LBW or PTB specifically among EA mothers residing in areas with elevated fish total mercury concentrations (Table 3). In a few cases, a statistically significant reduction in the risk of term LBW or PTB was observed, primarily among EA mothers in the intermediate total mercury exposure categories.

**Table 3:** **Relationship between adverse reproductive outcomes and estimated fish mercury exposure, stratified by race, all live births, South Carolina, 1995-2005**^**a**^

**Exposure estimate**	**Low birth weight**				**Preterm birth**			
	**European American**		**African American**		**European American**		**African American**	
	**(n = 218,319)**		**(n = 118,241)**		**(n = 217,187)**		**(n = 117,697)**	
	**OR**	**95% CI**	**OR**	**95% CI**	**OR**	**95% CI**	**OR**	**95% CI**
Predicted Mercury in Fish^b^								
Quartile 1	Ref	-	Ref	-	Ref	-	Ref	-
Quartile 2	1.01	0.96, 1.07	1.06	1.00, 1.13	1.06	1.02, 1.11	1.14	1.08, 1.21
Quartile 3	0.98	0.92, 1.04	1.13	1.07, 1.20	1.03	0.98, 1.08	1.18	1.11, 1.25
Quartile 4	0.96	0.91, 1.02	1.13	1.07, 1.20	0.96	0.92, 1.01	1.10	1.04, 1.17
Fish Advisory Categories								
<0.25 ppm	Ref	-	Ref	-	Ref	-	Ref	-
0.25-0.66 ppm	0.95	0.90, 1.00	1.10	1.05, 1.15	0.96	0.92, 1.00	1.12	1.07, 1.17
0.67-0.99 ppm	0.95	0.90, 1.01	1.08	1.02, 1.14	0.92	0.87, 0.97	1.01	0.95, 1.06
≥1.0 ppm	0.97	0.97, 1.07	1.18	1.07, 1.30	0.98	0.90, 1.07	1.13	1.02, 1.24
8-Kilometer Buffer Zones								
No restrictions	Ref	-	Ref	-	Ref	-	Ref	-
1 meal a week	0.88	0.82, 0.94	1.04	0.98, 1.10	0.96	0.91, 1.02	1.06	1.00, 1.12
1 meal a month	1.08	0.98, 1.19	1.14	1.06, 1.23	0.86	0.79, 0.94	0.86	0.80, 0.93
Do not eat	0.94	0.86, 1.02	1.15	1.08, 1.24	0.96	0.89, 1.03	0.98	0.92, 1.05

^a^Adjusted for: mother’s age, education, smoking status, the number of previous live births and stillborns. ^b^Based on kriged interpolation model, Q1: ND-0.17 ppm; Q2: >0.17-0.29 ppm; Q3: >0.29-0.62 ppm; Q4: >0.62 ppm. Q: quartile; OR: odds ratio; CI: confidence interval; ppm: parts per million. N’s are smaller than those presented in Table 1 due to missing data.

In ancillary analyses, the relationship between estimated fish total mercury exposures and term LBW or PTB was evaluated after stratification by race and either the Kotelchuck index or the mother’s education. Odds of term LBW among AA women with birth residences in the vicinity of elevated fish mercury was more likely to be observed among women with intermediate or adequate prenatal care compared to AA women with low estimated fish mercury exposures. No evidence for increased term LBW risk was observed among other combinations of exposure and prenatal care (Additional file 2: Table S2A), or for odds of PTB in conjunction with estimated fish mercury exposures among AA women stratified by prenatal care (Additional file 2: Table S2B). Similarly, no evidence of increased term LBW or PTB risk was observed among EA mothers evaluated in this manner (Additional file 2: Tables S2A and S2B). In some cases, a decreased likelihood of term LBW or PTB was observed among EA women, particularly among women with adequate-plus prenatal care (Additional file 2: Table S2A and S2B).

Results for analyses stratified by race and maternal education are provided as supplementary tables for term LBW (Additional file 3: Table S3A) and PTB (Additional file 3: Table S3B). Odds of LBW tended to be more common among AA mothers living in areas with elevated fish total mercury concentrations relative to those living in areas with low fish mercury exposure estimates regardless of mother’s educational status. When EA mothers were stratified by education, no consistent pattern of increased term LBW or PTB risk was observed in conjunction with estimated fish mercury exposures (Additional file 3: Tables S3A and S3B, respectively).

When SC ecoregions were evaluated, statistically significant increased odds of term LBW in relation to elevated fish total mercury exposure estimates were observed primarily in the Mid-Atlantic Coastal Plain counties (Berkeley, Dorchester, Florence, Hampton, Marion, and Williamsburg, Additional file 4: Table S4A). This region corresponds to counties directly inland from the coast but not adjacent to the coast, and it overlaps with some of the highest fish total mercury concentrations in SC (see Figure 1 and Additional file 5: Figure S1, SC county map). Also, there were fewer differences in exposure-response between AA and EA women in these counties relative to the statewide analysis.

## Discussion

This population-based, record-linkage study employed an EPHT framework that is useful for epidemiologic surveillance, risk screening, and resource prioritization [55]. The EPHT design provided a practical, cost effective strategy to explore possible associations between fish total mercury levels and adverse reproductive outcomes using readily available data; and the results can be used to help decide if more detailed studies may be warranted. The SC Vital Statistics Registry and fish biomonitoring program are valid and reliable resources that were useful for this purpose. The results suggest that women residing in SC regions with elevated total mercury concentrations in fish may have an increased likelihood of delivering a term LBW infant compared to women who live in areas with lower fish total mercury levels. These results are in agreement with several previous investigations of mercury and reproductive outcomes [37-39,42-45,47], although not all studies have been consistent [36-48]. This study is likely the first to examine this issue separately in a large population of AA women. When analyses were stratified by race, associations between predicted fish total mercury exposure and odds of term LBW were observed exclusively among AA mothers; the risk estimates were larger relative to the overall ORs, and they were more consistent among the different exposure estimates that were evaluated. Estimated LBW risks among AA mothers did not vary appreciably by maternal education, and they tended to be isolated within groups of women who received intermediate or adequate prenatal care. Thus, inadequate prenatal care, maternal education, or related factors are not likely to have influenced the results. Also, the analysis accounted for the effects of several major confounding factors, but not all known or suspected risk factors for LBW or PTB could be evaluated (e.g., access to health care or food sources, maternal stress, occupation, body mass index, alcohol or caffeine use, seasonal variation, exposure to air pollution or water disinfection by-products, or other social or behavioral factors). Such factors would have to exhibit the same spatial pattern as total fish mercury concentrations to exert a confounding effect. Nonetheless, the relatively low effect estimates and in some cases non-monotonic exposure-response trends limits interpretation of the results. The possibility that residual confounding led to spurious associations, or contributed to inconsistencies observed among the exposure estimates, or between LBW versus PTB, cannot be eliminated. Inconsistencies between the two study outcomes may have been influenced by differences in the precision and validity of birth weight quantification versus estimation of the gestational period (PTB), which can be less precise [38,58]. These inconsistencies also may suggest a specificity in the mechanism of action for mercury that differs between LBW and PTB. Inconsistencies or even protective effects have been observed in other studies of the relationship between prenatal mercury exposure and adverse reproductive, neurodevelopmental, or cognitive outcomes. These have been attributed to: potential differences in the timing or type of mercury exposure that was employed (e.g., cord blood versus maternal hair); a threshold effect or nonlinear dose–response relationship between mercury and health outcome; sex-specific effects; or a lack of adjustment for fish consumption [30]. The presence of residual toxins in fish other than mercury (e.g., polychlorinated biphenyls or PCBs), or the potential influence of beneficial nutrients (e.g., omega-3 fatty acids, selenium) also may have contributed to the inconsistencies observed in this and other studies [30]. PCB advisories are in place for some SC waterways, although the spatial overlap with mercury fish advisories is limited (http://www.scdhec.gov/fish/AdvisoryMap). Similarly, air pollution patterns tend to be concentrated near the major population centers and do not consistently match the statewide pattern of mercury in fish (http://www.scdhec.gov/HomeAndEnvironment/Air).

The present study combined individual-level reproductive health data with spatial exposure information, and the design has several notable strengths, limitations and uncertainties. The use of a publically available statewide sample over an eleven year span provided for low cost screening of potential fish mercury risks in SC with good statistical power and generalizability. Spatial and temporal variations in the distribution of total mercury in fish and the locations of consumption advisories assigned to waterways were assumed to remain relatively constant over time. Some temporal variation in SC fish total mercury levels has been reported [16,59], although ancillary analyses of the interaction between estimated total mercury exposure and year of birth did not alter the interpretation of the results (data not shown). Place of residence is a proxy for exposure that can introduce misclassification through various mechanisms. The duration of residence in a given household and changes in residence during pregnancy can influence fish mercury exposure. This uncertainty was at least partially addressed in the analysis via changes in exposure assignment that were based on changes in the birth address for a given mother if they occurred. However, some misclassification may have occurred if the gestational period did not fully coincide with the residence at birth. Approximately 9–32 percent of women change their residential location during pregnancy; mobility generally declines with socioeconomic status, and distances moved tend to be relatively short (median <10 km) [60]. These potential biases are likely to have been non-differential with respect to exposure assignment, which usually leads to an underestimation of risk. The identification of increased odds of term LBW in relation to areas with elevated total mercury in fish, despite the imprecision and possible non-differential exposure misclassification, suggests that more detailed, individual level studies may be warranted, particularly among AA women. When SC ecoregions were evaluated, statistically significant increased odds of LBW in relation to estimated fish mercury exposure tended to occur in the Mid-Atlantic Coastal Plain counties, an area where total mercury concentrations in fish are elevated compared to other areas in SC, which further supports this suggestion.

Individual fish consumption information was not available among mothers in this study. The degree to which pregnant women consumed locally caught fish, or were aware of local fish consumption advisories, thus represents a study uncertainty. Women may consume different amounts or types of fish, or fish from different regions (e.g., coastal, tidal, freshwater) or other (commercial) sources that have higher or lower mercury levels. Women living in areas with consumption warnings also may have limited their freshwater fish intake. However, one study among 3,015 reproductive age women in 12 states (including North Carolina) found that women who were aware of fish advisories consumed more fish meals annually than women who were unaware of a fish consumption advisory (48 versus 41 meals per year, respectively) [61]. Other studies indicate that awareness of regional fish consumption advisories tends to be low and that advisory awareness is not necessarily more common among higher-risk subgroups (e.g., pregnant women), nor does it always correspond to reduced consumption of high-mercury fish or lower mercury exposures [34,51]. These issues, as well as uncertainties associated with the estimation of acceptable intakes for mercury in fish, may also have contributed to the inconsistencies observed in the present study.

Fish consumption during pregnancy can elicit either beneficial or detrimental effects depending on behavior related to mercury exposure as well as other factors. Recently, there has been a national effort to reconcile this paradox. There is a growing concern that women curtail their fish intake due to concerns about mercury exposure [34,35,51]. The 2004 EPA/Food and Drug Administration (FDA) guidelines [62] advise women about fish consumption as it relates to potential mercury exposure but does not delve into the amount of fish that should be consumed to improve child health and development. In 2014, the EPA and FDA proposed an update to this guidance [63] to provide appropriate fish intakes for pregnant women, women who might become pregnant, or breastfeeding mothers that are consistent with national dietary guidelines (at least 8–12 ounces per week of a variety of fish lower in mercury)[64]. The draft update also identifies fish to be avoided due to elevated mercury levels (tilefish, shark, swordfish, king mackerel) and advises women to heed advisories for locally caught fish. The extent to which women can achieve a balance between the intake of polyunsaturated fatty acids and other beneficial nutrients in fish while avoiding the adverse neurodevelopmental or potential reproductive impacts of mercury has important public health implications both nationally and internationally, and emphasizes the need for culturally appropriate public health messaging and continued surveillance of mercury in commercial and local sources of fish caught for consumption.

Racial, ethnic and cultural differences in fish consumption are other important factors that contribute to possible mercury exposure [54]. Studies in a Virginia urban coastal community indicated that mean fish consumption rates among reproductive age AA women were consistently indicative of subsistence fishing behavior [50,65]. These observations are compatible with other studies indicating that fish consumption is greater among AA women [21,22,49-51,53,66], and that reproductive age AA women have elevated mercury exposures relative to EAs [21,22]. In the Florida panhandle, a study of hair mercury levels and fish consumption practices among reproductive age women indicated that only 31% were knowledgeable of the state’s consumption advisory, and that advisory awareness was lowest among AA women (20%) [19]. Pregnant women were less aware of Florida’s fish advisory for mercury than non-pregnant women, and hair mercury levels were higher among women who were unfamiliar with the advisory [19]. Another survey performed along the northeast Florida coast (Duval County) found that reproductive age AA women were among those with the highest fish consumption rates and hair mercury concentrations, and they had low awareness of fish consumption advisories relative to other race groups [66]. Studies conducted in SC indicated that most fishers ate their catch regardless of their knowledge of potential health risks [52], and that fish consumption was greater among AAs relative to EAs [52-54,67]. In a study that evaluated fishing behaviors and fish consumption rates among 258 people fishing the Savannah River [52,54], AAs exceeded EAs in mean number of fishing trips per month, mean number of fish meals per month, and mean fish portion size per meal [54]. Fish consumption rates were greater among AAs than EAs regardless of education level [54], and women and children ate caught fish as often as men [54]. In Charleston SC, 453 women attending the Medical University of SC Family Medicine Center or Women’s Health Center were surveyed to gauge awareness of the 2004 US EPA/FDA fish consumption advisory [68]. Overall, only 47% were aware of the advisory, and AAs were 85% less likely to be aware of the advisory than EAs [68]. The above summary suggests a consistent pattern of high fish consumption, low advisory awareness, and elevated mercury exposure among AA women of reproductive age in SC and in the southeastern US.

## Conclusions

It has been estimated that more than 300,000 children are born within the US each year with cord blood mercury levels exceeding the reference concentration defining safe intake (5.8 μg/L) [21]. Women living in US coastal areas tend to consume more fish and have elevated blood mercury levels compared to women dwelling in non-coastal areas [22]. Results from the present study suggest that residential proximity to SC waterways with elevated fish mercury may be related to increased term LBW risk, and that AA women may be particularly susceptible. However, the ecologic, cross-sectional nature of this investigation and the lack of individual fish consumption data are major sources of uncertainty that preclude establishment of a cause-effect relationship. The true prevalence of subsistence fishing in SC or other regionally similar areas is not known, and there is a paucity of dose–response information on the potential relationship between mercury intake and LBW or PTB. Quantifying mercury intake among susceptible individuals who are compelled to consume locally caught fish may be critical for optimizing public health interventions targeting mercury in rural SC, or in other vulnerable populations in the Southeast US or in international settings where subsistence fishing is prevalent.

## Abbreviations

AA: African-American; CI: Confidence interval; EA: European-American; GED: General education degree; GIS: Geographic information system; LBW: Low birth weight; OR: Odds ratio; PTB: Preterm birth; SC: South Carolina; SC DHEC: South Carolina Department of Health and Environmental Control; US: United States; USC: University of South Carolina.

## Competing interests

The authors declare that they have no competing interests.

## Authors’ contributions

JBB, ES, RP, BC, SWR and WK developed the study design and data analysis plan. SWR and RW developed the spatial model and GIS maps. SWR performed statistical analyses with input from JBB, BC, WK and other authors. JBB and SWR prepared the manuscript. RP, ES, WK, JV and BC provided peer review and consultation in specific areas of expertise. All authors read and approved the final manuscript.

## Supplementary Material

Additional file 1: Table S1 Relationship Between Adverse Reproductive Outcomes and Estimated Fish Mercury Exposure, Stratified by Race, First Births Only, South Carolina, 1995-2005. Click here for file

Additional file 2: Table S2 A. Low Birth Weight and Estimated Fish Mercury Exposure, Stratified by Race and Kotelchuck index All Live Births, South Carolina, 1995-2005^a^. B. Preterm Birth and Estimated Fish Mercury Exposure, Stratified by Race and Kotelchuck index, All Live Births, South Carolina, 1995-2005. Click here for file

Additional file 3: Table S3 A. Low Birth Weight and Estimated Fish Mercury Exposure, Stratified by Race and Mother’s Education. All Live Births, South Carolina, 1995-2005. Click here for file

Additional file 4: Table S4 A. Low Birth Weight and Estimated Fish Mercury Exposure, Stratified by Race and Ecoregion. All Live Births, South Carolina, 1995–2005 (N = 218,060). Click here for file

Additional file 5: Figure S1 South Carolina County Map. Click here for file
