# Supplementary material for: Mercury in fish and adverse reproductive outcomes: results from South Carolina
Source: Int J Health Geogr. 2014 Aug 15;13:30. doi: 10.1186/1476-072X-13-30 (PMC4154616; doi:10.1186/1476-072X-13-30)
Supplement: Additional file 1: Table S1 — Relationship Between Adverse Reproductive Outcomes and Estimated Fish Mercury Exposure, Stratified by Race, First Births Only, South Carolina, 1995-2005. [file 1476-072X-13-30-S1.docx]

| **Table S.1. Relationship Between Adverse Reproductive Outcomes and Estimated Fish Mercury Exposure, Stratified by Race, First Births Only, South Carolina, 1995-2005^a^** | | | | | | | | |
| --- | --- | --- | --- | --- | --- | --- | --- | --- |
| **Exposure Estimate** | **Low Birth Weight (N=150,988)** | | | | **Preterm Birth (N=150,253)** | | | |
|  | **European American** | | **African American** | | **European American** | | **African American** | |
|  | **OR** | **95% CI** | **OR** | **95% CI** | **OR** | **95% CI** | **OR** | **95% CI** |
| Predicted Mercury in Fish^b^ |  |  |  |  |  |  |  |  |
| Quartile 1 | Ref | - | Ref | - | Ref | - | Ref | - |
| Quartile 2 | 0.98 | 0.91, 1.05 | 1.09 | 1.00, 1.19 | 1.02 | 0.95, 1.08 | 1.17 | 1.07, 1.28 |
| Quartile 3 | 0.94 | 0.87, 1.02 | 1.14 | 1.05, 1.24 | 1.01 | 0.94, 1.08 | 1.15 | 1.05, 1.25 |
| Quartile 4 | 0.92 | 0.85, 0.99 | 1.12 | 1.03, 1.22 | 0.91 | 0.85, 0.97 | 1.08 | 0.99, 1.18 |
| Fish Advisory Categories |  |  |  |  |  |  |  |  |
| <0.25 ppm | Ref | - | Ref | - | Ref | - | Ref | - |
| 0.25-0.66 ppm | 0.92 | 0.86, 0.98 | 1.08 | 1.02, 1.15 | 0.95 | 0.90, 1.01 | 1.07 | 1.01, 1.14 |
| 0.67-0.99 ppm | 0.94 | 0.86, 1.01 | 1.05 | 0.97, 1.13 | 0.91 | 0.85, 0.97 | 0.96 | 0.89, 1.04 |
| >1.0 ppm | 0.86 | 0.74, 0.99 | 1.22 | 1.07, 1.41 | 0.89 | 0.79, 1.01 | 1.33 | 1.15, 1.53 |
| 8-Kilometer Buffer Zones |  |  |  |  |  |  |  |  |
| No restrictions | Ref | - | Ref | - | Ref | - | Ref | - |
| 1 meal a week | 0.85 | 0.78, 0.93 | 1.03 | 0.94, 1.12 | 0.93 | 0.87, 1.01 | 0.98 | 0.90, 0.92 |
| 1 meal a month | 1.05 | 0.92, 1.19 | 1.14 | 1.02, 1.26 | 0.84 | 0.74, 0.95 | 0.82 | 0.74, 0.92 |
| Do not eat | 0.89 | 0.79, 1.00 | 1.23 | 1.11, 1.35 | 0.92 | 0.84, 1.02 | 1.00 | 0.91, 1.11 |
| ^a^ Adjusted for: mother’s age, education, smoking status, and number of previous live births and stillborns.  ^b^ Based on kriged interpolation model, Q1: ND-0.17 ppm; Q2: >0.17-0.29 ppm; Q3: >0.29-0.62 ppm; Q4: >0.62 ppm. Q: quartile; OR: odds ratio; CI: confidence interval; ppm: parts per million. | | | | | | | | |
