# Supplementary material for: Mercury in fish and adverse reproductive outcomes: results from South Carolina
Source: Int J Health Geogr. 2014 Aug 15;13:30. doi: 10.1186/1476-072X-13-30 (PMC4154616; doi:10.1186/1476-072X-13-30)
Supplement: Additional file 2: Table S2 — A. Low Birth Weight and Estimated Fish Mercury Exposure, Stratified by Race and Kotelchuck index All Live Births, South Carolina, 1995-2005a. B. Preterm Birth and Estimated Fish Mercury Exposure, Stratified by Race and Kotelchuck index, All Live Births, South Carolina, 1995-2005. [file 1476-072X-13-30-S2.doc]

| **Table S.2.A. Low Birth Weight and Estimated Fish Mercury Exposure, Stratified by Race and Kotelchuck index, All Live Births, South Carolina, 1995-2005^a^** | | | | | | | | | | |
| --- | --- | --- | --- | --- | --- | --- | --- | --- | --- | --- |
| **Exposure Estimate** | **Kotelchuck Index Category** | | | | | | | | | |
|  | **Inadequate**  **(n=48,267)** | | | **Intermediate**  **(n=41,936)** | | | **Adequate**  **(n=129,825)** | | **Adequate plus**  **(n=113,654)** | |
|  | **OR** | **95% CI** | | **OR** | | **95% CI** | **OR** | **95% CI** | **OR** | **95% CI** |
| **Predicted Mercury in Fish^b^** | **European American** | | | | | | | | | |
| Quartile 1 | Ref | - | | Ref | | - | Ref | - | Ref | - |
| Quartile 2 | 1.05 | 0.90, 1.21 | | 0.88 | | 0.71, 1.08 | 1.00 | 0.89, 1.13 | 0.92 | 0.86, 0.99 |
| Quartile 3 | 0.99 | 0.84, 1.17 | | 1.01 | | 0.80, 1.27 | 0.89 | 0.78, 1.01 | 0.87 | 0.81, 0.95 |
| Quartile 4 | 0.91 | 0.79, 1.06 | | 0.96 | | 0.77, 1.19 | 1.06 | 0.94, 1.19 | 0.81 | 0.75, 0.88 |
|  | **African American** | | | | | | | | | |
| Quartile 1 | Ref | - | | Ref | | - | Ref | - | Ref | - |
| Quartile 2 | 1.10 | 0.98, 1.24 | | 0.90 | | 0.73, 1.10 | 1.08 | 0.95, 1.23 | 0.98 | 0.90, 1.08 |
| Quartile 3 | 1.05 | 0.94, 1.18 | | 1.23 | | 1.01, 1.49 | 1.15 | 1.01, 1.31 | 1.01 | 0.93, 1.10 |
| Quartile 4 | 1.03 | 0.92, 1.15 | | 1.18 | | 0.97, 1.43 | 1.25 | 1.09, 1.42 | 1.02 | 0.94, 1.12 |
| **Fish Advisory Categories** | **European American** | | | | | | | | | |
| <0.25 ppm | Ref | - | | Ref | | - | Ref | - | Ref | - |
| 0.25-0.66 ppm | 0.96 | 0.83, 1.09 | | 0.95 | | 0.78, 1.15 | 0.91 | 0.82, 1.01 | 0.87 | 0.82, 0.93 |
| 0.67-0.99 ppm | 0.82 | 0.70, 0.95 | | 1.04 | | 0.83, 1.29 | 1.08 | 0.96, 1.22 | 0.88 | 0.81, 0.95 |
| >1.0 ppm | 1.05 | 0.82, 1.34 | | 0.78 | | 0.49, 1.24 | 0.94 | 0.74, 1.20 | 0.78 | 0.68, 0.90 |
|  | **African American** | | | | | | | | | |
| <0.25 ppm | Ref | - | | Ref | | - | Ref | - | Ref | - |
| 0.25-0.66 ppm | 1.03 | 0.94, 1.12 | | 1.21 | | 1.04, 1.41 | 1.08 | 0.98, 1.19 | 1.04 | 0.97, 1.11 |
| 0.67-0.99 ppm | 0.94 | 0.85, 1.04 | | 1.29 | | 1.08, 1.54 | 1.19 | 1.06, 1.34 | 1.04 | 0.96, 1.12 |
| >1.0 ppm | 1.08 | 0.90, 1.30 | | 1.30 | | 0.92, 1.83 | 1.27 | 1.02, 1.58 | 1.02 | 0.89, 1.17 |
| **8-Kilometer Buffer Zones** | **European American** | | | | | | | | | |
| No restrictions | Ref | - | | Ref | - | | Ref | - | Ref | - |
| 1 meal a week | 0.87 | 0.73, 1.03 | | 0.73 | 0.56, 0.96 | | 0.86 | 0.75, 0.99 | 0.85 | 0.78, 0.93 |
| 1 meal a month | 1.14 | 0.89, 1.47 | | 0.94 | 0.67, 1.32 | | 1.26 | 1.03, 1.53 | 0.97 | 0.85, 1.11 |
| Do not eat | 0.76 | 0.61, 0.95 | | 0.79 | 0.58, 1.09 | | 1.06 | 0.89, 1.27 | 0.88 | 0.78, 0.98 |
|  | **African American** | | | | | | | | | |
| No restrictions | Ref | - | | Ref | - | | Ref | - | Ref | - |
| 1 meal a week | 1.05 | 0.93, 1.18 | | 1.01 | 0.80, 1.28 | | 1.00 | 0.87, 1.15 | 1.00 | 0.92, 1.09 |
| 1 meal a month | 1.12 | 0.97, 1.29 | | 1.42 | 1.11, 1.83 | | 1.29 | 1.09, 1.52 | 0.96 | 0.86, 1.06 |
| Do not eat | 1.12 | 0.98, 1.28 | | 1.51 | 1.18, 1.92 | | 1.32 | 1.13, 1.54 | 1.00 | 0.91, 1.11 |
| ^a^ Adjusted for: mother’s age, education, smoking status, number of previous live births and stillborns. ^b^ Based on kriged interpolation model, Q1: ND-0.17 ppm; Q2: >0.17-0.29 ppm; Q3: >0.29-0.62 ppm; Q4: >0.62 ppm. Q: quartile; OR: odds ratio; CI: confidence interval; ppm: parts per million. | | | | | | | | | | |
| **Table S.2.B. Preterm Birth and Estimated Fish Mercury Exposure, Stratified by Race and Kotelchuck index, All Live Births, South Carolina, 1995-2005^a^** | | | | | | | | | | |
| **Exposure Estimate** | **Kotelchuck Index Category** | | | | | | | | | |
|  | **Inadequate**  **(n=48,267)** | | | **Intermediate**  **(n=41,936)** | | | **Adequate**  **(n=129,825)** | | **Adequate plus**  **(n=113,654)** | |
|  | **OR** | **95% CI** | | **OR** | **95% CI** | | **OR** | **95% CI** | **OR** | **95% CI** |
| **Predicted Mercury in Fish^b^** | **European American** | | | | | | | | | |
| Quartile 1 | Ref | - | | Ref | - | | Ref | - | Ref | - |
| Quartile 2 | 1.12 | 0.98, 1.27 | | 0.92 | 0.75, 1.13 | | 0.93 | 0.83, 1.05 | 0.96 | 0.91, 1.02 |
| Quartile 3 | 1.13 | 0.98, 1.30 | | 0.79 | 0.62, 1.01 | | 0.91 | 0.80, 1.03 | 0.89 | 0.83, 0.94 |
| Quartile 4 | 0.98 | 0.86, 1.11 | | 0.92 | 0.74, 1.14 | | 0.86 | 0.76, 0.98 | 0.83 | 0.78, 0.88 |
|  | **African American** | | | | | | | | | |
| Quartile 1 | Ref | - | | Ref | - | | Ref | - | Ref | - |
| Quartile 2 | 1.14 | 1.02, 1.28 | | 1.00 | 0.80, 1.26 | | 1.16 | 0.99, 1.35 | 1.08 | 0.99, 1.17 |
| Quartile 3 | 1.09 | 0.98, 1.22 | | 1.15 | 0.92, 1.43 | | 1.10 | 0.95, 1.28 | 1.07 | 0.99, 1.16 |
| Quartile 4 | 1.00 | 0.89, 1.11 | | 1.09 | 0.87, 1.36 | | 1.08 | 0.93, 1.26 | 1.04 | 0.96, 1.13 |
| **Fish Advisory Categories** | **European American** | | | | | | | | | |
| <0.25 ppm | Ref | - | | Ref | - | | Ref | - | Ref | - |
| 0.25-0.66 ppm | 1.02 | 0.90, 1.14 | | 0.77 | 0.63, 0.94 | | 0.93 | 0.84, 1.03 | 0.86 | 0.82, 0.90 |
| 0.67-0.99 ppm | 0.91 | 0.79, 1.04 | | 0.96 | 0.77, 1.19 | | 0.88 | 0.78, 1.01 | 0.87 | 0.81, 0.92 |
| >1.0 ppm | 1.06 | 0.86, 1.32 | | 0.92 | 0.59, 1.42 | | 0.78 | 0.60, 1.02 | 0.76 | 0.68, 0.85 |
|  | **African American** | | | | | | | | | |
| <0.25 ppm | Ref | - | | Ref | - | | Ref | - | Ref | - |
| 0.25-0.66 ppm | 1.07 | 0.99, 1.17 | | 1.15 | 0.97, 1.37 | | 1.08 | 0.96, 1.21 | 1.03 | 0.97, 1.10 |
| 0.67-0.99 ppm | 0.91 | 0.82, 1.00 | | 1.13 | 0.92, 1.38 | | 1.00 | 0.87, 1.15 | 0.99 | 0.92, 1.07 |
| >1.0 ppm | 1.01 | 0.85, 1.21 | | 1.04 | 0.68, 1.58 | | 0.99 | 0.75, 1.31 | 1.00 | 0.88, 1.14 |
| **8-Kilometer Buffer Zones** | **European American** | | | | | | | | | |
| No restrictions | Ref | - | Ref | | - | | Ref | - | Ref | - |
| 1 meal a week | 1.10 | 0.95, 1.28 | 0.91 | | 0.70, 1.17 | | 0.80 | 0.70, 0.92 | 0.92 | 0.86, 0.98 |
| 1 meal a month | 0.87 | 0.68, 1.10 | 0.64 | | 0.43, 0.96 | | 0.88 | 0.71, 1.10 | 0.82 | 0.74, 0.92 |
| Do not eat | 0.93 | 0.77, 1.12 | 0.93 | | 0.68, 1.28 | | 0.90 | 0.75, 1.08 | 0.88 | 0.80, 0.95 |
|  | **African American** | | | | | | | | | |
| No restrictions | Ref | - | Ref | | - | | Ref | - | Ref | - |
| 1 meal a week | 1.07 | 0.96, 1.20 | 0.84 | | 0.65, 1.08 | | 0.99 | 0.85, 1.16 | 1.05 | 0.97, 1.14 |
| 1 meal a month | 0.82 | 0.71, 0.94 | 0.89 | | 0.67, 1.19 | | 0.91 | 0.74, 1.12 | 0.76 | 0.69, 0.84 |
| Do not eat | 0.87 | 0.77, 0.99 | 0.89 | | 0.67, 1.18 | | 1.14 | 0.95, 1.36 | 0.95 | 0.86, 1.05 |
| ^a^ Adjusted for: mother’s age, education, smoking status, number of previous live births and stillborns. OR: odds ratio; CI: confidence interval; ppm: parts per million. ^b^ Based on kriged interpolation model, Q1: ND-0.17 ppm; Q2: >0.17-0.29 ppm; Q3: >0.29-0.62 ppm; Q4: >0.62 ppm. Q: quartile; OR: odds ratio; CI: confidence interval; ppm: parts per million. | | | | | | | | | | |
