# Supplementary material for: Mercury in fish and adverse reproductive outcomes: results from South Carolina
Source: Int J Health Geogr. 2014 Aug 15;13:30. doi: 10.1186/1476-072X-13-30 (PMC4154616; doi:10.1186/1476-072X-13-30)
Supplement: Additional file 3: Table S3 — A. Low Birth Weight and Estimated Fish Mercury Exposure, Stratified by Race and Mother’s Education. All Live Births, South Carolina, 1995-2005. [file 1476-072X-13-30-S3.doc]

| **Table S.3.A. Low Birth Weight and Estimated Fish Mercury Exposure, Stratified by Race and Mother’s Education, All Live Births, South Carolina, 1995-2005^a^** | | | | | | |
| --- | --- | --- | --- | --- | --- | --- |
| **Exposure Estimate** | **Mother’s Educational Status** | | | | | |
|  | **11^th^ Grade or Less**  **(n=70,756)** | | **High School or GED**  **(n=113,861)** | | **College**  **(n=152,696)** | |
|  | **OR** | **95% CI** | **OR** | **95% CI** | **OR** | **95% CI** |
| **Predicted Mercury in Fish^b^** | **European American** | | | | | |
| Quartile 1 | Ref | - | Ref | - | Ref | - |
| Quartile 2 | 0.95 | 0.86, 1.05 | 0.97 | 0.89, 1.07 | 1.10 | 1.01, 1.21 |
| Quartile 3 | 0.94 | 0.83, 1.06 | 0.93 | 0.83, 1.03 | 1.06 | 0.96, 1.16 |
| Quartile 4 | 0.89 | 0.79, 0.99 | 0.94 | 0.86, 1.05 | 1.02 | 0.93, 1.12 |
|  | **African American** | | | | | |
| Quartile 1 | Ref | - | Ref | - | Ref | - |
| Quartile 2 | 1.06 | 0.95, 1.17 | 1.06 | 0.96, 1.16 | 1.07 | 0.95, 1.21 |
| Quartile 3 | 1.11 | 1.01, 1.23 | 1.11 | 1.01, 1.22 | 1.17 | 1.05, 1.31 |
| Quartile 4 | 1.11 | 1.00, 1.23 | 1.10 | 1.01, 1.21 | 1.19 | 1.07, 1.34 |
| **Fish Advisory Categories** | **European American** | | | | | |
| <0.25 ppm | Ref | - | Ref | - | Ref | - |
| 0.25-0.66 ppm | 0.95 | 0.86, 1.05 | 0.92 | 0.84, 1.00 | 0.98 | 0.91, 1.05 |
| 0.67-0.99 ppm | 0.91 | 0.81, 1.03 | 0.94 | 0.85, 1.04 | 0.98 | 0.89, 1.08 |
| >1.0 ppm | 0.81 | 0.66, 0.99 | 1.02 | 0.87, 1.21 | 1.02 | 0.86, 1.22 |
|  | **African American** | | | | | |
| <0.25 ppm | Ref | - | Ref | - | Ref | - |
| 0.25-0.66 ppm | 1.10 | 1.02, 1.19 | 1.07 | 1.00, 1.14 | 1.15 | 1.05, 1.24 |
| 0.67-0.99 ppm | 1.07 | 0.97, 1.17 | 1.04 | 0.96, 1.13 | 1.15 | 1.04, 1.26 |
| >1.0 ppm | 1.13 | 0.96, 1.33 | 1.20 | 1.04, 1.37 | 1.20 | 0.97, 1.49 |
| **8-Kilometer Buffer Zones** | **European American** | | | | | |
| No restrictions | Ref | - | Ref | - | Ref | - |
| 1 meal a week | 0.78 | 0.69, 0.90 | 0.93 | 0.83, 1.04 | 0.90 | 0.81, 0.99 |
| 1 meal a month | 1.05 | 0.88, 1.25 | 1.06 | 0.90, 1.25 | 1.11 | 0.94, 1.31 |
| Do not eat | 0.85 | 0.73, 1.00 | 0.98 | 0.85, 1.13 | 0.95 | 0.83, 1.09 |
|  | **African American** | | | | | |
| No restrictions | Ref | - | Ref | - | Ref | - |
| 1 meal a week | 1.03 | 0.92, 1.15 | 1.03 | 0.94, 1.14 | 1.04 | 0.94, 1.16 |
| 1 meal a month | 1.12 | 0.98, 1.28 | 1.15 | 1.03, 1.28 | 1.11 | 0.97, 1.27 |
| Do not eat | 1.12 | 0.99, 1.28 | 1.13 | 1.02, 1.25 | 1.20 | 1.06, 1.37 |
| ^a^ Adjusted for: mother’s age, smoking status, number of previous live births and stillborns. ^b^ Based on kriged interpolation model, Q1: ND-0.17 ppm; Q2: >0.17-0.29 ppm; Q3: >0.29-0.62 ppm; Q4: >0.62 ppm. Q: quartile; OR: odds ratio; CI: confidence interval; GED: general equivalency diploma; ppm: parts per million. | | | | | | |

| **Table S.3.B. Preterm Birth and Estimated Fish Mercury Exposure, Stratified by Race and Mother’s Education, All Live Births, South Carolina, 1995-2005^a^** | | | | | | |
| --- | --- | --- | --- | --- | --- | --- |
| **Exposure Estimate** | **Mother’s Educational Status** | | | | | |
|  | **11^th^ Grade or Less**  **(n=70,365)** | | **High School or GED**  **(n=113,254)** | | **College**  **(n=151,991)** | |
|  | **OR** | **95% CI** | **OR** | **95% CI** | **OR** | **95% CI** |
| **Predicted Mercury in Fish^b^** | **European American** | | | | | |
| Quartile 1 | Ref | - | Ref | - | Ref | - |
| Quartile 2 | 1.02 | 0.93, 1.12 | 1.08 | 1.00, 1.17 | 1.08 | 1.01, 1.16 |
| Quartile 3 | 1.01 | 0.90, 1.13 | 1.04 | 0.95, 1.13 | 1.03 | 0.96, 1.11 |
| Quartile 4 | 0.98 | 0.88, 1.08 | 0.97 | 0.89, 1.06 | 0.95 | 0.88, 1.02 |
|  | **African American** | | | | | |
| Quartile 1 | Ref | - | Ref | - | Ref | - |
| Quartile 2 | 1.21 | 1.09, 1.35 | 1.12 | 1.02, 1.23 | 1.09 | 0.97, 1.22 |
| Quartile 3 | 1.19 | 1.07, 1.32 | 1.18 | 1.08, 1.29 | 1.16 | 1.04, 1.29 |
| Quartile 4 | 1.17 | 1.05, 1.30 | 1.04 | 0.95, 1.14 | 1.10 | 0.99, 1.23 |
| **Fish Advisory Categories** | **European American** | | | | | |
| <0.25 ppm | Ref | - | Ref | - | Ref | - |
| 0.25-0.66 ppm | 0.97 | 0.89, 1.07 | 0.97 | 0.90, 1.04 | 0.94 | 0.89, 1.00 |
| 0.67-0.99 ppm | 0.98 | 0.88, 1.09 | 0.91 | 0.83, 0.99 | 0.90 | 0.83, 0.96 |
| >1.0 ppm | 0.91 | 0.76, 1.09 | 1.01 | 0.87, 1.16 | 1.00 | 0.87, 1.14 |
|  | **African American** | | | | | |
| <0.25 ppm | Ref | - | Ref | - | Ref | - |
| 0.25-0.66 ppm | 1.11 | 1.03, 1.20 | 1.11 | 1.04, 1.19 | 1.14 | 1.05, 1.24 |
| 0.67-0.99 ppm | 1.07 | 0.97, 1.18 | 0.94 | 0.87, 1.02 | 1.04 | 0.95, 1.15 |
| >1.0 ppm | 1.06 | 0.90, 1.25 | 1.16 | 1.01, 1.33 | 1.16 | 0.95, 1.42 |
| **8-Kilometer Buffer Zones** | **European American** | | | | | |
| No restrictions | Ref | - | Ref | - | Ref | - |
| 1 meal a week | 1.00 | 0.88, 1.13 | 0.96 | 0.87, 1.06 | 0.94 | 0.88, 1.02 |
| 1 meal a month | 0.89 | 0.75, 1.06 | 0.84 | 0.73, 0.97 | 0.85 | 0.75, 0.98 |
| Do not eat | 0.94 | 0.81, 1.08 | 0.99 | 0.88, 1.11 | 0.94 | 0.84, 1.04 |
|  | **African American** | | | | | |
| No restrictions | Ref | - | Ref | - | Ref | - |
| 1 meal a week | 1.06 | 0.95, 1.18 | 1.10 | 1.00, 1.20 | 1.00 | 0.91, 1.11 |
| 1 meal a month | 0.81 | 0.71, 0.93 | 0.92 | 0.82, 1.03 | 0.83 | 0.73, 0.95 |
| Do not eat | 0.96 | 0.85, 1.09 | 1.03 | 0.93, 1.14 | 0.93 | 0.82, 1.05 |
| ^a^ Adjusted for: mother’s age, smoking status, number of previous live births and stillborns. ^b^ Based on kriged interpolation model, Q1: ND-0.17 ppm; Q2: >0.17-0.29 ppm; Q3: >0.29-0.62 ppm; Q4: >0.62 ppm. Q: quartile; OR: odds ratio; CI: confidence interval; GED: general equivalency diploma; ppm: parts per million. | | | | | | |
