# Supplementary material for: Mercury in fish and adverse reproductive outcomes: results from South Carolina
Source: Int J Health Geogr. 2014 Aug 15;13:30. doi: 10.1186/1476-072X-13-30 (PMC4154616; doi:10.1186/1476-072X-13-30)
Supplement: Additional file 4: Table S4 — A. Low Birth Weight and Estimated Fish Mercury Exposure, Stratified by Race and Ecoregion. All Live Births, South Carolina, 1995–2005 (N = 218,060). [file 1476-072X-13-30-S4.docx]

| **Table S.4.A. Low Birth Weight and Estimated Fish Mercury Exposure, Stratified by Race and Ecoregion**  **All Live Births, South Carolina, 1995-2005 (N=218,060)^a^** | | | | | | | |
| --- | --- | --- | --- | --- | --- | --- | --- |
| **Exposure Estimate** | **Ecoregion^b^** | | | | | | |
|  | **Southern Plains**  **(n=108,936)** | | **Mid-Atlantic Coastal Plain**  **(n=40,669)** | | | **Coastal Region**  **(n=68,455)** | |
|  | **OR** | **95% CI** | **OR** | | **95% CI** | **OR** | **95% CI** |
| **Predicted Mercury in Fish** | **European American** | | | | | | |
| Quartile 1 | Ref | - | Ref | | - | Ref | - |
| Quartile 2 | 1.04 | 0.78, 1.40 | 1.34 | | 1.07, 1.68 | 0.61 | 0.28, 1.31 |
| Quartile 3 | 1.14 | 0.87, 1.51 | 1.31 | | 1.04, 1.64 | 0.66 | 0.61, 1.42 |
| Quartile 4 | 1.12 | 0.85, 1.47 | 1.44 | | 1.15, 1.80 | 0.67 | 0.31, 1.43 |
|  | **African American** | | | | | | |
| Quartile 1 | Ref | - | Ref | | - | Ref | - |
| Quartile 2 | 0.91 | 0.72, 1.16 | 1.05 | | 0.81, 1.34 | 1.97 | 0.96, 4.04 |
| Quartile 3 | 1.02 | 0.81, 1.28 | 1.20 | | 0.95, 1.53 | 1.91 | 0.93, 3.91 |
| Quartile 4 | 0.96 | 0.76, 1.21 | 1.36 | | 1.10, 1.70 | 1.94 | 0.94, 3.96 |
| **Fish Advisory Categories** | **European American** | | | | | | |
| <0.25 ppm | Ref | - | | Ref | - | Ref | - |
| 0.25-0.66 ppm | 1.13 | 0.98, 1.29 | | 1.05 | 0.91, 1.22 | 0.92 | 0.75, 1.13 |
| 0.67-0.99 ppm | 1.16 | 1.01, 1.34 | | 1.17 | 0.99, 1.39 | 0.92 | 0.74, 1.13 |
| >1.0 ppm | 0.80 | 0.50, 1.29 | | 1.04 | 0.71, 1.52 | 1.09 | 0.88, 1.35 |
|  | **African American** | | | | | | |
| <0.25 ppm | Ref | - | | Ref | - | Ref | - |
| 0.25-0.66 ppm | 1.13 | 1.03, 1.25 | | 1.19 | 1.01, 1.40 | 0.99 | 0.88, 1.11 |
| 0.67-0.99 ppm | 1.08 | 0.97, 1.19 | | 1.25 | 1.07, 1.47 | 0.96 | 0.84, 1.11 |
| >1.0 ppm | 0.96 | 0.64, 1.43 | | 1.43 | 1.16, 1.76 | 1.05 | 0.90, 1.22 |
| **8-Kilometer Buffer Zones** | **European American** | | | | | | |
| No restrictions | Ref | - | | Ref | - | Ref | - |
| 1 meal a week | 0.95 | 0.83, 1.09 | | 1.02 | 0.81, 1.28 | 0.78 | 0.48, 1.29 |
| 1 meal a month | 1.12 | 0.96, 1.31 | | 1.42 | 1.10, 1.83 | 0.63 | 0.23, 1.75 |
| Do not eat | 0.99 | 0.84, 1.16 | | 1.05 | 0.75, 1.48 | 0.91 | 0.55, 1.50 |
|  | **African American** | | | | | | |
| No restrictions | Ref | - | | Ref | - | Ref | - |
| 1 meal a week | 1.00 | 0.91, 1.10 | | 1.33 | 1.02, 1.73 | 1.05 | 0.68, 1.62 |
| 1 meal a month | 1.04 | 0.94, 1.16 | | 1.53 | 1.18, 1.98 | 1.75 | 0.96, 3.18 |
| Do not eat | 1.05 | 0.95, 1.17 | | 1.63 | 1.24, 2.15 | 1.19 | 0.77, 1.85 |
| ^a^ Adjusted for: mother’s age, smoking status and number of previous live births and stillborns. ^b^Ecoregion designations: Southern Plains: Aiken, Allendale, Bamberg, Calhoun, Chesterfield, Clarendon, Edgefield, Darlington, Dillon, Kershaw, Lee, Lexington, Marlboro, Orangeburg, Richland, Sumter; Mid-Atlantic Coastal Plain: Berkeley, Dorchester, Florence, Hampton, Marion, Williamsburg; Coastal Region: Beaufort, Charleston, Colleton, Georgetown, Horry, Jasper. Q1: ND-0.17 ppm; Q2: >0.17-0.29 ppm; Q3: >0.29-0.62 ppm; Q4: >0.62 ppm. Q: quartile; OR: odds ratio; CI: confidence interval; GED: general equivalency diploma; ppm: parts per million. | | | | | | | |

| **Table S.4.B. Preterm Birth and Estimated Fish Mercury Exposure, Stratified by Race and Ecoregion**  **All Live Births, South Carolina, 1995-2005 (N=217,618)^a^** | | | | | | | | |
| --- | --- | --- | --- | --- | --- | --- | --- | --- |
| **Exposure Estimate** | **Ecoregion^b^** | | | | | | | |
|  | **Southern Plains**  **(n=108,752)** | | **Mid-Atlantic Coastal Plain**  **(n=40,575)** | | | **Coastal Region**  **(n=68,291)** | | |
|  | **OR** | **95% CI** | **OR** | **95% CI** | | **OR** | **95% CI** | |
| **Predicted Mercury in Fish** | **European American** | | | | | | | |
| Quartile 1 | Ref | - | Ref | - | | Ref | - | |
| Quartile 2 | 1.17 | 0.93, 1.48 | 1.24 | 1.04, 1.47 | | 0.80 | 0.39, 1.65 | |
| Quartile 3 | 1.17 | 0.93, 1.46 | 1.22 | 1.03, 1.45 | | 0.81 | 0.39, 1.66 | |
| Quartile 4 | 1.14 | 0.91, 1.42 | 1.06 | 0.89, 1.26 | | 0.84 | 0.41, 1.71 | |
|  | **African American** | | | | | | | |
| Quartile 1 | Ref | - | Ref | - | Ref | | - | |
| Quartile 2 | 0.98 | 0.77, 1.25 | 1.05 | 0.82, 1.34 | 1.96 | | 1.01, 3.83 | |
| Quartile 3 | 1.11 | 0.88, 1.41 | 1.19 | 0.95, 1.50 | 1.82 | | 0.93, 3.56 | |
| Quartile 4 | 1.06 | 0.84, 1.34 | 1.11 | 0.90, 1.38 | 1.73 | | 0.88, 3.38 | |
| **Fish Advisory Categories** | **European American** | | | | | | | |
| <0.25 ppm | Ref | - | Ref | - | Ref | | | - |
| 0.25-0.66 ppm | 0.99 | 0.89, 1.11 | 1.00 | 0.89, 1.12 | 0.92 | | | 0.78, 1.10 |
| 0.67-0.99 ppm | 1.03 | 0.92, 1.15 | 0.93 | 0.81, 1.07 | 0.88 | | | 0.74, 1.04 |
| >1.0 ppm | 0.96 | 0.67, 1.37 | 1.02 | 0.75, 1.37 | 1.08 | | | 0.90, 1.29 |
|  | **African American** | | | | | | | |
| <0.25 ppm | Ref | - | Ref | - | Ref | | | - |
| 0.25-0.66 ppm | 1.15 | 1.05, 1.27 | 1.06 | 0.91, 1.25 | 1.02 | | | 0.91, 1.13 |
| 0.67-0.99 ppm | 1.06 | 0.95, 1.17 | 1.03 | 0.88, 1.21 | 0.89 | | | 0.78, 1.02 |
| >1.0 ppm | 1.10 | 0.76, 1.60 | 1.31 | 1.07, 1.60 | 0.97 | | | 0.83, 1.12 |
| **8-Kilometer Buffer Zones** | **European American** | | | | | | | |
| No restrictions | Ref | - | Ref | - | Ref | | | - |
| 1 meal a week | 0.89 | 0.80, 0.99 | 1.14 | 0.95, 1.36 | 1.90 | | | 1.07, 3.36 |
| 1 meal a month | 0.80 | 0.70, 0.91 | 0.98 | 0.79, 1.22 | 1.07 | | | 0.40, 2.85 |
| Do not eat | 0.87 | 0.76, 0.98 | 1.32 | 1.03, 1.69 | 1.62 | | | 0.91, 2.86 |
|  | **African American** | | | | | | | |
| No restrictions | Ref | - | Ref | - | Ref | | | - |
| 1 meal a week | 0.99 | 0.91, 1.08 | 1.04 | 0.81, 1.33 | 1.14 | | | 0.74, 1.75 |
| 1 meal a month | 0.72 | 0.65, 0.80 | 0.95 | 0.75, 1.20 | 1.60 | | | 0.88, 2.92 |
| Do not eat | 0.79 | 0.71, 0.87 | 1.20 | 0.93, 1.55 | 1.14 | | | 0.74, 1.75 |
| ^a^ Adjusted for: mother’s age, smoking status and number of previous live births and stillborns. ^b^Ecoregion designations: Southern Plains: Aiken, Allendale, Bamberg, Calhoun, Chesterfield, Clarendon, Edgefield, Darlington, Dillon, Kershaw, Lee, Lexington, Marlboro, Orangeburg, Richland, Sumter; Mid-Atlantic Coastal Plain: Berkeley, Dorchester, Florence, Hampton, Marion, Williamsburg; Coastal Region: Beaufort, Charleston, Colleton, Georgetown, Horry, Jasper. Q1: ND-0.17 ppm; Q2: >0.17-0.29 ppm; Q3: >0.29-0.62 ppm; Q4: >0.62 ppm. Q: quartile; OR: odds ratio; CI: confidence interval; GED: general equivalency diploma; ppm: parts per million. | | | | | | | | |
