# Supplementary figures and images for: Mercury in fish and adverse reproductive outcomes: results from South Carolina
Source: Int J Health Geogr. 2014 Aug 15;13:30. doi: 10.1186/1476-072X-13-30 (PMC4154616; doi:10.1186/1476-072X-13-30)

Figure S1. South Carolina County Map


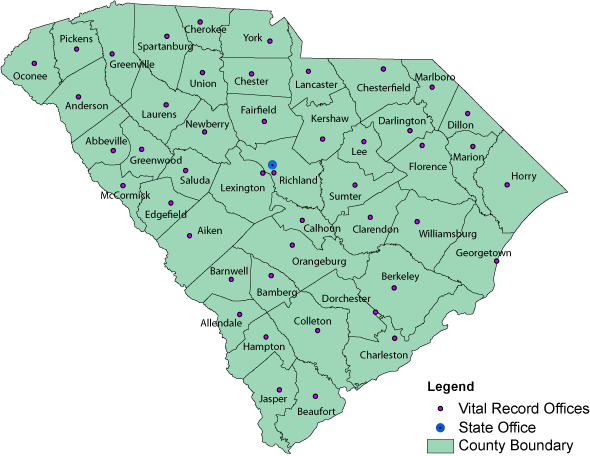

Supplement: Additional file 5: Figure S1 — South Carolina County Map. [file 1476-072X-13-30-S5.docx]
